# Advancement on Lead-Free Organic-Inorganic Halide Perovskite Solar Cells: A Review

**DOI:** 10.3390/ma11061008

**Published:** 2018-06-14

**Authors:** Faruk Sani, Suhaidi Shafie, Hong Ngee Lim, Abubakar Ohinoyi Musa

**Affiliations:** 1 Department of Physics, Usmanu Danfodiyo University, P.M.B. 2346, Sokoto, Nigeria; 2 Functional Devices Laboratories, Institute of Advanced Technology, Universiti Putra Malaysia, Serdang 43400, Malaysia; suhaidi@upm.edu.my; 3 Faculty of Engineering, Universiti Putra Malaysia, Serdang 43400, Malaysia; 4 Department of Chemistry, Faculty of Science, Universiti Putra Malaysia, Serdang 43400, Malaysia; hongngee@upm.edu.my; 5 Materials Synthesis and Characterization Laboratory, Institute of Advanced Technology, Universiti Putra Malaysia, Serdang 43400, Malaysia; 6 Department of Physics, Bayero University, Gwarzo Road, 700241 Kano, Nigeria; aomusa.phy@buk.edu.ng

**Keywords:** halides, inorganic cation, lead free perovskite, solar cells, anions, methyl-ammonium

## Abstract

Remarkable attention has been committed to the recently discovered cost effective and solution processable lead-free organic-inorganic halide perovskite solar cells. Recent studies have reported that, within five years, the reported efficiency has reached 9.0%, which makes them an extremely promising and fast developing candidate to compete with conventional lead-based perovskite solar cells. The major challenge associated with the conventional perovskite solar cells is the toxic nature of lead (Pb) used in the active layer of perovskite material. If lead continues to be used in fabricating solar cells, negative health impacts will result in the environment due to the toxicity of lead. Alternatively, lead free perovskite solar cells could give a safe way by substituting low-cost, abundant and non toxic material. This review focuses on formability of lead-free organic-inorganic halide perovskite, alternative metal cations candidates to replace lead (Pb), and possible substitutions of organic cations, as well as halide anions in the lead-free organic-inorganic halide perovskite architecture. Furthermore, the review gives highlights on the impact of organic cations, metal cations and inorganic anions on stability and the overall performance of lead free perovskite solar cells.

## 1. Introduction

**Figure 1:**
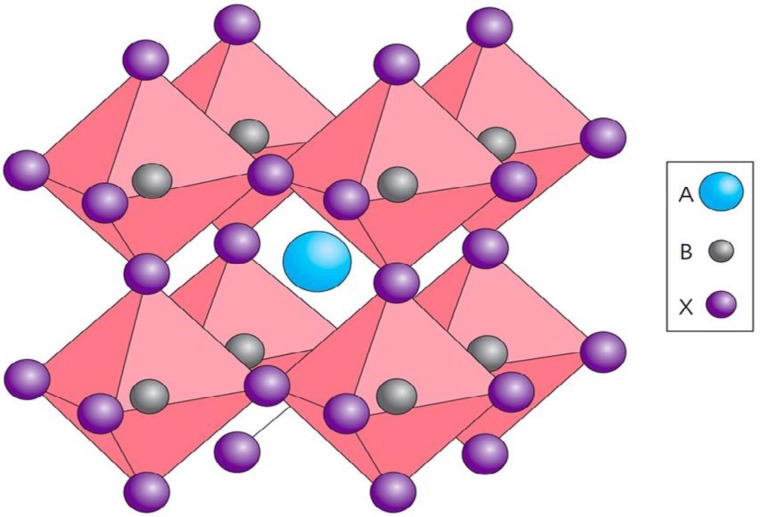
A typical crystal structure of a Perovskite [6].

Organic inorganic halide perovskite solar cells have shown significant improvement of power conversion efficiency (PCE) from the initial efficiency of 3.8% [1] to about 22% [2]. The highest theoretical power conversion efficiency achieved by perovskite (CH_3_NH_3_PbI_3_) is 31.4% [3]. This remarkable performance is achieved due to unique properties of organic-halide perovskite to exhibit ambipolar transport, high absorption co-efficient, charges carrier mobility, long diffusion length, direct and tunable band gap, simple methods of fabrication [4,5,6]. Furthermore, the cell is composed of earth-abundant material and attracted tremendous attention in the photovoltaic research field [7]. Perovskite generally refers to any crystalline material with chemical formula ABX_3_ similar to CaTiO_3_ [8] as shown in Figure 1.

Most of the materials served as a photo-active layer in perovskite solar cells are comprised of an organo-metal halide. Typically, organic-cation (A) include (methyl-ammonium ‘MA’, formamidiu ‘FA’) with a range of divalent metal cations (B) being utilized such as Cu^2+^, Fe^2+^, Mn^2+,^ Pb^2+^, Sn^2+^, etc. These cations have been mixed with halide anions (X) include F^−^, Cl^−^, Br^−^ and I^−^. Combining A-cation, B-cation and X-anion different ratios, various perovskite materials can be obtained [9]. Direct substitution either of the cations or anions may results in distorted perovskite structure or non-perovskite. Goldschmidt Tolerance Factor (GTF) is a dimensionless empirical index which can predict regular crystal structure of a perovskite [10]. GTF is calculated using an expression;

(1) $$t=\frac{r_{A+r_{X}}}{\sqrt{2}\;\left(r_{B}+r_{X}\right)}$$

where $r_{A}$ is an ionic radius of A-cation, $r_{B}$ is an ionic radius of B-cation, and $r_{X}$ is an ionic radius of X-anion.

Generally, tolerance factor, t, of 0.9–1.0 has an ideal cubic structure. Tolerance factor, *t*, less than 0.71 or greater than 1.0 resulted in forming non-perovskite structure [11]. When *t* < 0.8, indicates that A-cation is very small and this might distort the formation of perovskite structure, and when *t* > 1, the A-cation is very big and this also might result in the formation of non-perovskite. Commonly perovskite structure can be formed in the range, 0.8 ≤ *t* ≤ 1.0. High tolerance factor limit for hybrid iodide perovskite is between1.06 and 1.07 [12]. In the case of hybrid perovskite due to organic cations and hydrogen bond, GTF cannot absolutely predict their ionic radius. To solve this complexity, Cheetham and co-workers applied rigid sphere model for organic cations and assumed rotational freedom and extended the Goldschmidt Tolerance Factor equation to

(2) $$t=\frac{r_{A,eff+r_{X}}}{\sqrt{2}\;\left(r_{B}+r_{X}\right)}$$

$r_{A,eff}$ is given as $r_{mass}+r_{ion}$ [13]. The major concern issue for perovskite-based solar cells is the used of heavy metal lead (Pb) in the light absorbing layer. World Health Organization (WHO) has declared lead as an undesirable element causing major societal negative health impacts. Thus there is need for collective effort to protect the health of people in all categories, regardless of age or gender. The Institute for Health Metrics and Evaluation (IHME) estimated that Pb ingestion is responsible for 9.3% of world intellectual disorder, 4.0% of the world problem of heart diseases and 6.6% of the world problem of stroke [14]. Therefore, photovoltaic community conducted a lot of research aiming to overcome the toxicity challenge in perovskite based solar cells by substituting Pb with other elements to find an environmentally friendly absorber layer for perovskite solar cells [15]. Recently, many researchers are committed to discover further possible lead-free perovskite absorber material and to modify the device architecture, giving rise to momentous advancement in power conversion efficiency (PCE) of a lead-free organic-inorganic halide perovskite solar cell [8]. This review discusses the recent advancement of lead-free organic-inorganic halide perovskite solar cells. It also includes the experimental results, theoretical results of lead-free perovskite solar cells, and focuses on different possible substitutions in the three components of the lead free perovskite absorber layer; organic cations, metal cations and halide anions.

## 2. Structure of Lead-Free Organic-Inorganic Hybrid Halide Perovskites

Lead-free organic-inorganic halide perovskite is class of materials that have the same chemical formula ABX_3_. In an ideal lead-free organic-inorganic halide perovskite, the A^+^ cation is at the corners of a cube, the X^−^ anion is in the middle of each faces and the small B^+^ (lead free) cation is in the middle of the octahedral sites formed by the anions as shown in Figure 1 [9]. Where A is organic cation (such as ammonium, [NH_4_]^+^, hydroxyl-ammonium, [CH_3_OH]^+^, methyl-ammonium, [CH_3_NH_3_]^+^, formamidinium, [CH(NH_2_)_2_]^+^, ethylammonium, [CH_3_CH_2_)NH_3_]^+^ etc.) and B is inorganic cation, like group 14 element (e.g., Sn^2+^, Ge^2+^), alkaline earth metals (such as Mg^2+^, Ca^2+^ etc.), transition divalent metals (such as Cu^2+^, Zn^2+^ etc.), and lanthanide (e.g., Eu^2+^, Yb^2+^).Each B^+^ cation mentioned can be served as an alternative to replace divalent lead cation (Pb^2+^) [15]. Perovskite absorber layer consisting Germanium, Silicon, Tin or mixture of either two cations are most suitable in terms of overall photovoltaic performance to replace lead cation [16]. Germanium and Silicon are the best alternatives to lead [17]. These alternative elements (Si^2+^, Sn^2+^ and Ge^2+^) are unstable on exposure to air [18,19,20]. X is an anion in the formula and are halogens (F^−^, Cl^−^, Br^−^ and I^−^) or ([HCOO]^−^, [CN]^−^, [BH_4_]^−^), SCN^−^ [13,16]. The main functions of the inorganic sites are to ensure stability and structural order, while the organic component give the mechanical flexibility and cost effective processing [21] and the X anions maintained the charge neutrality between cations and anions [8]. The stability of organic-inorganic halide perovskite depends on the requirements of Goldschmidt Tolerance Factor. The calculated effective ionic radii of several A-cation, B-cations and X-anion are shown in Table 1.

**Figure 2:**
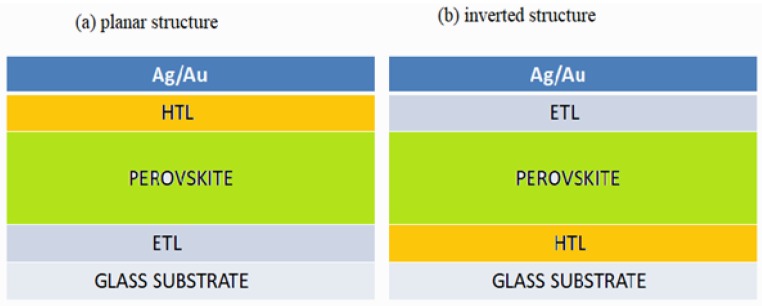
(**a**) A planar device architecture; (**b**) An inverted device architecture.

Typically, there are two major device architectures for organo-metal halide perovskite structure without considering the mesoscopic nano-material; planar heterojuction (n-i-p) and inverted structure (p-i-n) [24]. For planar heterojunction, the electron transporting layer (ETL) is spin cast on a glass substrate (e.g., Fluorine-doped tin oxide (FTO)/glass or Indium tin oxide (ITO)/glass followed by perovskite photo-active layer, hole-transporting material (HTL), and finally a metal contact (e.g., silver or gold). In the inverted planar architecture, the hole-transporting material (HTL) is first deposited on a glass substrate then deposition of perovskite layer, electron transporting layer (ETL), and finally a metal contact as shown in the schematic diagram of inverted structure and conventional structure in Figure 2a,b respectively. The most reported inverted planar heterojunction device is PEDOT:PSS [poly (ethylenedioxythiophene): poly (styrene sulfonate)]/perovskite layer/PCBM (phenl-C61-butyric acid methyl ester). In this device, PEDOT:PSS and PCBM function as HTL and ETL respectively. The inverted structure exhibits significant power conversion efficiency (PCE) and virtually free-hysteresis in the voltage-current output, easy fabrication method, and cost effectiveness [25,26]. For conventional planar configuration, using metal-oxide (such as TiO_2_, SnO_2_ or ZnO) as the electron-transport layer (ETL), the device could show high performance under reverse scan (open-circuit voltage to short circuit current scan). Poor electron injection and extraction exhibited in p-i-n perovskite structure might be due to a barrier at the contact interface between Fermi level of the metal electrode and the lowest un-occupied molecular orbit of the ETM. That is why currently inverted structure is not yet achieved comparable power conversion efficiency to regular planar perovskite architecture [27]. Planar architecture can be further simplified by removing an expensive hole transporting material (HTM) commonly spiro-OMeTAD into a new design called planar HTM-fee architecture [28]. Planar structure with HTM-free is also a promising direction towards reducing the fabrication cost and enhancing the efficiency and stability of perovskite solar cells. Reference [29] designed a planar HTM-free based solar cells and the device exhibited relative stability. This directed towards a potential application of cost effective solar devices.

## 3. Organic Cation Substitution

**Figure 3:**
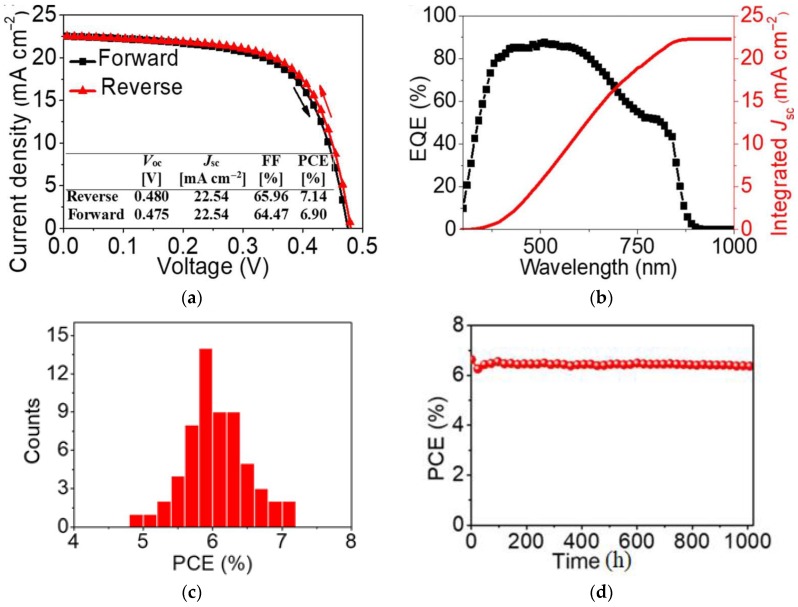
(**a**) Current density-Voltage (Jsc-V) of the cell {en}FASnI_3_ perovskite absorber with 10% en loading under reverse and forward voltage scan; (**b**) EQE and integrated Jsc measured from solar cells with 10% en; (**c**) Histograms of power conversion efficiencies (PCEs) for 60% solar cells with 10%; (**d**) The efficiency of an encapsulated device with 10% en as a function of the storage time. Reproduced with permission [41]. Copyright American Asociation for the Advancement of Science, 2017.

Organic cation in ABX_3_ plays a significant role in the structural formation of perovskite structures and also has a great effect on the stability and opto-electronic properties of the perovskite material [30]. A cation substitution should strictly base on BX_6_ octahedral sharing with the respect to a Goldschmidt tolerance factor. A-cation substitution is aimed to obtain more-stable and appropriate dynamic position of the conduction band of the perovskite film [31]. The most extensively studied organic cations in lead free organo metal halide perovskite are methyl-ammonium (MA) and formadinium (FA). Dimentionality and stability of perovskite lattice could be significantly affected by the sizes and functionality of the A-cation [8]. Physical properties such as light absorption and charge transport might also be affected by methyl-ammonium ions (NH_3_CH_3_^+^) and formamidinium ions (CH(NH_2_)_2_^+^) [9]. Mixing MA and FA cations show a remarkable performance in the morphology of lead free based perovskite and reduction of charge carrier recombination using (FA)_0.75_(MA)_0.25_ SnI_3_ device structure using 10 mol % SnF_2_ additive and this achieved a power conversion efficiency (PCE) of 8.12% [32]. Use of volatile cation such as MA^+^ and FA^+^ might be responsible for instability in lead-free organic inorganic halide perovskite. Reference [33] employed Cs^+^ in place of methyl-ammonium and formamidinium and resulted in high tolerance to the environment. Cs^+^ in lead-free organic inorganic halide perovskite exhibited stability better than the device with the same structural arrangement using methyl-ammonium lead-iodide perovskite [34]. Lead-free organic-inorganic halide perovskite employed MA or Cs as cation have a smaller size than the same device used FA as an organic cation. The unit cell in FASnI_3_ has a larger size than the MASnI_3_ and CsSnI_3_ and their smaller size resulted in chemical pressure on the inorganic lattice [35]. The differences in size of A-organic cations play the influential role in the stability of perovskite solar cell. In 2016, reference [36] explored a lead-free perovskite solar cell where Cs^+^ is added SnF_2_ and replaced MA^+^ in cesium tin bromide (CsSnBr_3_) serves as an active layer in the n-i-p structure. The fabricated solar cell achieved power conversion efficiency (PCE) of 2.1% and this is better than the reported CsSnBr_3_-based solar cells. This might be due to the incorporation of tin fluoride (SnF_2_) as suggested by the author. Combined small amount of Cs cation in MA based perovskite structure, improves the photovolaic performance but reduces the cell’s stability due to the mixture of two different organic cations with different ionic radius [37]. Another combined Cs cation and Rb cation was reported by [38] in CsRbSn_2_I_6_ lead free absorber layer. The solar cell displays an octahedral rotation pattern and semi-conducting band gap capable of light absorption. The most important remark on Cs^+^ is its owing property of stability improvement in lead free perovskite solar cells. It shows remarkable stability improvement in tin based perovskite when exposed to ambient air [39]. The substitution of Cs^+^ with MA^+^ or FA^+^ resulted in the increase in the volume of the perovskite and hence the band gap increased [40]. Ethylenediammonium (en) as an organic cation improves the air stability and increases the power conversion efficiency (PCE) of lead free organic-inorganic perovskite to 7.14% in {en}FASnI_3_ structure Figure 3 [41]. 2-phenylethyl alcohol (PEAl) is recently introduced as an organic cation in tin-based perovskite solar cells. The presence of PEAl in the device improved the PCE to 6.98% [42]. This will add another stepping block towards achieving high efficient lead-free perovskite solar cells. Defect tolerance of lead-free perovskites is related to the lattice polarizability and does not require larger organic cation such methyl-ammonium or formamidinium [43]. It was observed that the octahedra deformation increases by the increase in an ionic radius of organic cation [44]. Furthemore, A-cation substitution were found to be responsible for expansion, contraction, or octahedral tilting of the perovskite framework and this could also directly or indirectly affect the bandgap and optical properties of the material [45].

In the struggle for searching suitable organic cations, Goldschmidts tolerance factor, *t*, should be given proper attention for finding essential organic cation such as Hydroxylammonium (216 pm), Hydrazinium (217 pm), 3-pyrollinium (272 pm), Thiazolium (320 pm), Guanidinium (278 pm), and potassium [13,22]. The crystal lattice of perovskites expands with the increase in ionic radius of the organic cation (Cesium < methyl-ammonium < formamidinium) [30]. 

## 4. Inorganic Cation Substitution

### 4.1. Tin Halides

**Figure 4:**
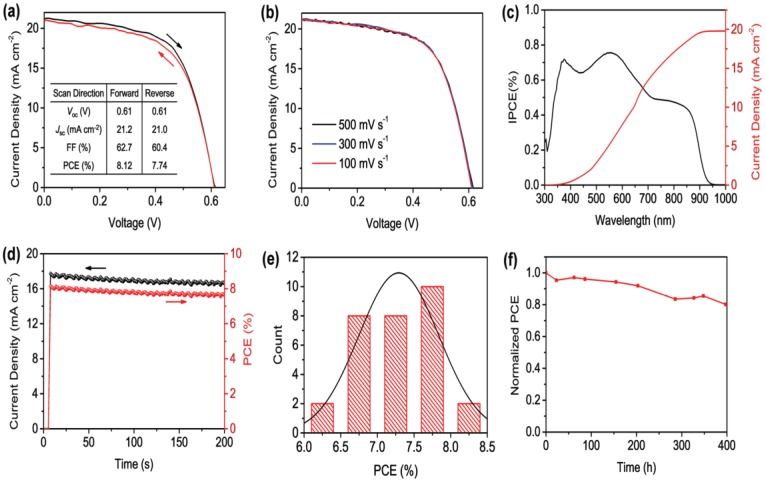
(**a**) J-V curves of the champion device measured using both forward and reverse scan mode at rate of 300 mV/s under the simulation of A.M 1.5 G, 100 mW/cm^2^; (**b**) J-V curves of the champion drvice measured at different scan rates using forward scan mode under the simulation of A.M 1.5 G, 100 mW/cm^2^; (**c**) Incident photon-to-current efficiency (IPCE) spectrum of the encapsulated (FA)_0.75_(MA)_0.25_SnI_3_- based device (**d**) steady state Jsc and PCE of the (FA)_0.75_(MA)_0.25_SnI_3_ measured at a bias of 0.46 V under AM 1.5 G, 100 mW/cm^2^ irradiation (**e**) PCE histogram of 30 (FA)_0.75_(MA)_0.25_SnI_3_-based device from several fabrication batches; (**f**) Normalized PCE of a (FA)_0.75_(MA)_0.25_SnI_3_-based device stored in glovebox over a period of 400 h. Reproduced with permission [32]. Copyright-WILEY-VCH Verlag, GmbH & Co. KGaA, Weinheim, Germany, 2017.

**Figure 5:**
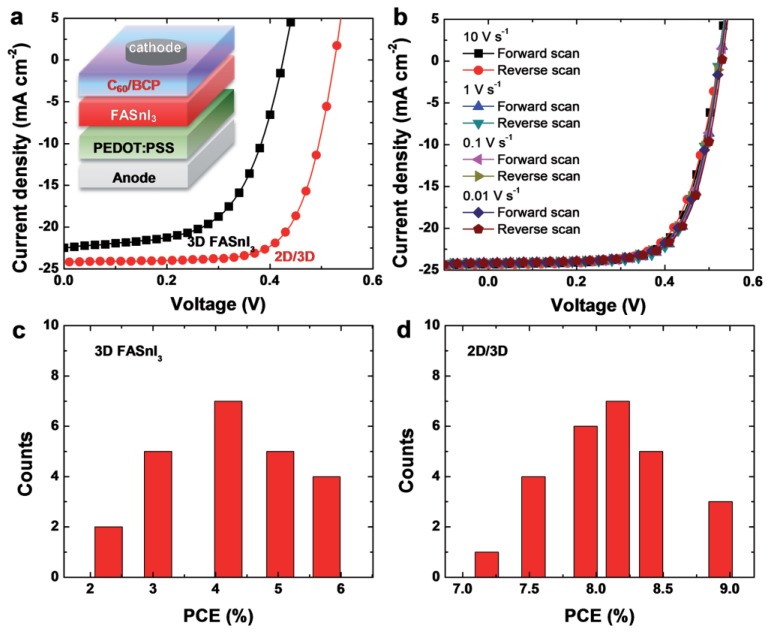
Device structure and characterization. (**a**) J-V curves under one sun AM 1.5 G condition for the champion devices containing pure 3D and 2D (0.008 m)/3D perovskite (the inset shows the device structure); (**b**) forward and reverse sweeps of the J-V characteristics of the champion 2D/3D perovskite cell measured at different rates; (**c**) histogram of the reference cell reproducibility, and (**d**) of the 2D/3D perovskite devices. Reproduced with permission [51]. Copyright-WILEY-VCH Verlag, GmbH & Co. KGaA, Weinheim, Germany, 2018.

To achieve Pb-free perovskite solar cells, Sn^2+^ metal cation was the first divalent metal used as an alternative candidate to replace Pb^2+^ because of its similar electronic configuration and close effective ionic radius (Sn^2+^: 115 pm) to lead (Pb^2+^: 119 pm) [8,10]. Sn^2+^ has a tendency to oxidize into the Sn^4+^ state, and this oxidation process might distort the neutrality between cations and anions in the perovskite structure [15,19,41,46]. The first reported tin-based perovskite absorber is fabricated by Noel et al. with a structure; FTO/comp-TiO_2_/mesoporousTiO_2_/CH_3_NH_3_SnI_3_/Spiro-OMeTAD/Au. The planar structure fetched power conversion efficiency (PCE) of 6.4%. To avoid exposure to air, the entire fabrication was carried out inside a glove box. CH_3_NH_3_SnI_3_ is a promising candidate to be a light sensitizer with suitable inorganic hole-transport material to achieve cost effective and efficient lead free perovskite solar cell. Reference [47], designed a Sn-based perovskite simulated model and analyzed the photovoltaic performance in planar structure; Glass/ZnO/Al/TiO_2_/CH_3_NH_3_SnI_3_/CuI/Au using Solar Cell Capacitance Simulator (SCAPS-D1) software. The structure predicts short circuit current of 25.67 mA/cm^2^, fill factor of 78.14%, open circuit voltage of 1.0413 V and power conversion efficiency of about 24.82% [47]. Reference [36] tackled the issue of the rapid oxidation process of tin when exposed to ambient air by adding SnF_2_ onto CsSnBr_3_. It was observed that SnF_2_ significantly improved the stability of the solar cell and power conversion efficiency of 2.1% was realized. Excess SnF_2_ functioned as an inhibitor of Sn^4+^ in FASnI_3_ based absorber layer and 4.8% PCE is obtained [48]. For the investigation to enhance the stability of tin-based perovskite, reference [49] reported the synthesized CsSnBr_3_-based perovskite by selecting precursors and temperature with improved stability. Further optimization using formamidinium (FA) and methyl-ammonium (MA) mixed cations into tin iodide perovskite forming (FA)_0.75_ (MA)_0.25_ SnI_3_ yielded high open circuit voltage of 0.61 V, short circuit current of 21.2 mA/cm^2^, fill factor of 64.6% and exhibited power conversion efficiency of 8.12% [32]. Figure 4 shows the photovoltaic performance of the tin-based perovskite solar cells. Reference [50] controlled crystallization and film formation of CH_3_NH_3_SnI_3_ by dissolving equimolar quantities of methyl-ammonium iodide (CH_3_NH_3_I) and tin iodide (SnI_2_) in dimethyl-formamide (DMF) and dimethyl-sulfoxide (DMSO) with gamma-butyrolactone (GBL) in a ratio 3.5:7 respectively. The results illustrate that DMF-based film shows higher surface roughness (*R*_rms_ = 1.85 nm), good crystallinity and better excited charge carrier extraction in contrast to DMSO-GBL film. This proves that crystallinity and film formation of Sn-based perovskite solar cell can be controlled by choosing a suitable precursor solvent. Further experimental results have recently revealed that mixing small quantity of 2-D PEA_2_SnI_4_ layered with 3D FASnI_3_ perovskite enhance the morphological and orientation of the 3D FASnI_3_ grain, resulting in a mixture of 2D/3D perovskite. The planar structure; ITO/PEDOT:PSS/FASnI_3_/C60 + BCP/Al shows high Voc of 0.525 V, Jsc of 24.1 mA/cm^2^, FF of 0.71, and PCE of 9.0% [51]. This is the currently highest PCE reported in a lead-free organic-inorganic halide perovskite solar cell. The device performance is shown in Figure 5.

Moreover, a simulated study using SCAP-1D on CH_3_NH_3_SnI_3_ based solar cell involving varios hole transport material (HTM) layers (spiro-OMeTAD, Cu_2_O, CuSCN). Among the three different structure, ZnO/CH_3_NH_3_SnI_3_/Cu_2_O/Au structure predicts the best photovoltaic performance by yielding open circuit voltage (0.85 V), short circuit current (32.26 mA/cm^2^), fill-factor (74.02%), and power conversion efficiency (20.23%) [52]. This structure could be a potential architecture for efficient and inexpensive lead free perovskite solar cells.

### 4.2. Germanium Halides

Another group 14 element called germanium is also potential metal suitable to substitute lead in perovskite solar cells [8,53]. Germanium halide perovskites are rarely reported in photovoltaic applications due its oxidation process, relatively small ionic radius and its poor solubility in polar solvents result to too wide band gap (>1.6 eV) and appalling morphology with very low power conversion efficiency (PCE) of only 0.2% from soluton process [54]. Methyl-ammonium, CH_3_NH_3_GeI_3_, formamidinium, HC[NH_2_]_2_GeI_3_, acetamidinium, CH_3_C[NH_2_]_2_GeI_3_, guanidinium, C[NH_2_]GeI_3_, trimethyl-ammonium, [CH_3_]_3_NHGeI_3_ and isopropylammonium [CH_3_]_2_C[H]NH_3_GeI_3_ were synthesized, analysed and concluded that MAGeI_3_ and FAGeI_3_ show the best photovoltaic performance [55]. Introducing 10% bromide ions additive into methyl-ammonium germanium iodide perovskite in p-i-n architecture; ITO/glass/PEDOT:SS/MAGe_2.7_Br_0.3_/PC_70_BM/Ag improves the power conversion efficiency (PCE) to 0.57% along with a slight development in the stability of germanium based perovskite solar cell [20]. Mixing cation strategy predicts potentiality of RbSn_0.5_Ge_0.5_I_3_ as an active layer in lead free perovskite, owing to its direct band gap (0.9–1.6 eV), light absorption which is similar to MAPbI_3_, high charge carrier mobility and moisture resistance [56].

### 4.3. Bismuth Halides Perovskite

**Figure 6:**
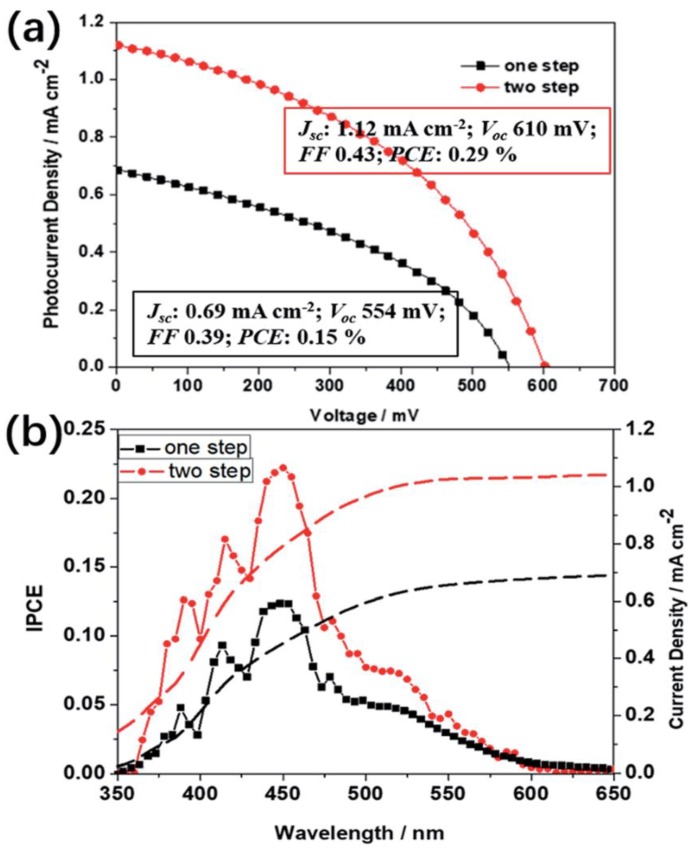
(**a**) J-V curves for the best performing devices by the one-step and the two-step method, respectively, measured under simulated AM1.5 G irradiation; (**b**) Corresponding IPCEs. Re-used with permission from [64]. Copyright, Royal Society of Chemistry, 2017.

Bismuth is a group 15 metal capable of replacing lead in perovskite solar cells. Bi^3+^ is an isoelectronic to Pb^2+^ having the same 6 s^2^ lone pair, analogous electro-negativity (Bi: 2.02 and Pb: 2.33) and possesses effective ionic radius equivalent to divalent lead ions (Bi^3+^ = 103 pm and Pb2 = 119 pm) [8]. Organic-inorganic bismuth halides perovskite attracted attention due to stability in ambient atmosphere, less/non-toxic nature and simple method of processing [57]. The early reported bismuth halide perovskites include methyl-ammonium bismuth iodide (MBI) in planar structure; FTO/_comp_-TiO_2_/mesosp-TiO_2_/MBI/P3HT/Au. This device displayed poor photocurrent performance and thus yielded very low power conversion efficiency of −0.190% [57]. This could be due to indirect large band gap. To improve the photocurrent conversion as well as reducing the band gap in bismuth based perovskites, [58] introduced Cs^+^ cation in place of MA^+^ in CsBi_3_I_10_ chemical structure as a light harvester in the perovskite solar devices. The author also compared the device with the previous reported A_3_Bi_2_I_9_ (A: methyl-ammonium or cesium) structure by [59]. CsBi_3_I_10_ yields 1.77 eV bandgap which is lower than 2.03 eV band-gap obtained using Cs_3_Bi_2_I_9_ and improves the photocurrent up to 700 nm with low PCE of 0.40%. Calculated bandgaps based on Density Functional Theory (DFT) calculation found direct/indirect band gap of 2.17 eV/2.0 eV and 2.17 eV/1.97 eV for Cs_3_Bi_2_I_9_ and MA_3_Bi_2_I_9_ respectively which are tallied with the experimental band-gap values of 2.2 eV, 2.0 eV and 2.4 eV, 2.1 eV for Cs_3_Bi_2_I_9_ and MA_3_Bi_2_I_9_respectively [60]. In a search for the bismuth halide improvement, optical and structural properties of CH_3_NH_3_BiI_3_ were observed. The results show crystalline structure and band gap, −2 eV and <0.01% PCE [61]. Bismuth halide perovskites are stable in ambient air but unfortunately, too large band gap could be responsible for their lower power conversion efficiency. BiI_3_ is a promising light absorber because of its good optical indirect band gap (1.57 eV) capable of absorbing sunlight in photovoltaic applications [62]. The major challenge in using BiI_3_ as an active material perovskite solar cell is the mismatched alignment of energy level between BiI_3_ and TiO_2_. In this regard, neither single BiI_3_ nor A_3_Bi_2_I_9_ is suitable for efficient absorber in lead-free perovskite solar cells. Thus, reference [63], prepared active layers of BiI_3_ and A_3_Bi_2_I_9_ using chemical solution method under ambient temperature. The composite solar cell significantly increases the open circuit voltage from 0.44 V to 0.57 V and power conversion efficiency (PCE) from 0.045% to 0.076%. One step deposition technique was repeatedly adopted for depositing MBI, in which both BiI_2_ and methyl-ammonium iodide (MAI) dissolved in a common solvent such as DMF and spin cast on a glass substrate, the fabricated device, may result in poor morpholgy, homogeneity and low film coverage. This might be the factor hindering the good photovoltaic performance of bismuth-based perovskite. Further study towards high efficient bismuth halide absorber in perovskite solar cells revealed that two step method where BiI_3_ and CH_3_NH_3_I were spin coated separately on a mesoporous TiO_2_ resulted in an increase in PCE to 0.29% compared with the previous reported PCE [64]. Figure 6 shows the PCE and IPCE of the device using two-step technique by [64].

### 4.4. Copper Halide Perovskite

Copper is one of the earth abundant elements, it is non-toxic and possesses high charge mobility [65,66]. The divalent Cu^2+^ cation is also another alternative element for Pb^2+^ substitution in perovskite solar cells. Cu^2+^ is more stable in ambient air than Sn^2+^ and Ge^2+^ [54]. Cu^2+^ has smaller effective ionic radius (73 pm) compared to Pb^2+^ (119 pm). This hinders the formation 3D perovskite structure. Thus, copper-based halide perovskite form 2D layered structure type in (R-NH_3_)_2_MX_4_ chemical formula. R-NH_3_^+^ are either aliphatic or aromatic ammonium cation, X are halogens or pseudohalogens, and M can be monovalent, divalent or trivalent metal ions. The structures are body centered tetragonal and consist of layers of corner sharing MX_6_ octahedral with R-NH_3_ cations occupying holes between the X-anions on either side of the layers [66]. 2D cupric bromide halide perovskite was prepared as a light sensitizer in solar cells. The measured photovoltaic performances of the fabricated device are Jsc, Voc, FF, and PCE with the values; 1.78 mA/cm^2^, 0.88 V, 0.40, and 0.63% respectively [66]. Stability of copper halide perovskite could be greatly improved by mixed halides. It has been observed that small quantity of Cl^−^ enhanced crystallization and stability without affecting the photovoltaic performance. Using this development, reference [67] investigated and characterized (CH_3_NH_3_)_2_CuCl_4_, (CH_3_NH_3_)_2_CuCl_2_Br_2_, (CH_3_NH_3_)_2_CuClBr_3_, and (CH_3_NH_3_)_2_CuCl_0.5_Br_3.5_. The band gap and the respective absorption coefficient of the absorbers were determined as follows; in MA_2_CuCl_4_ (2.48 eV and 500 nm)_,_ in (CH_3_NH_3_)_2_CuCl_2_Br_2_ (2.12 eV and 584 nm) for (CH_3_NH_3_)_2_CuClBr_3_ (1.90 eV and 625 nm), in (CH_3_NH_3_)_2_CuCl_0.5_Br_3.5_ (1.80 eV and 689 nm)_._ (CH_3_NH_3_)_2_CuCl_2_Br_2_ and (CH_3_NH_3_)_2_CuCl_0.5_Br_3.5_ have shown better stability. The highest PCE of 0.017% is obtained using MA_2_CuCl_2_Br_2_. The author suggested that the increased in green photoluminescence intensity is due to Cu^2+^ reduction to +1 state. This emission is applicable to light emitting devices. Moreover, the effort to conquer the challenges of Pb-based perovskite is extended to the idea of double perovskite with the general formula; A_2_(BC)X_6_ where 2Pb in APbX_3_ is transmuted into a metal pair (BC) such as Cu/Ag or Ga/In. This made Cu-based the leading thin film photovoltaic absorber. The new absorber Rb_2_(CuIn)Cl_6_ shows a required band gap of 1.36 eV and strong light absorption [68]. The alternative method of double perovskite by incorporating a combination of metals is recently attracting the photovoltaic community towards achieving efficient, non-toxic, cost effective and stable perovskite solar cells. Copper (Cu) and antimony (Sb) are synthesized in CsCuSb_2_Cl_12_ absorber. The Cu-Sb based perovskite exhibit a direct band gap of 1.0 eV and good conductivity compared to MAPbI_3_. Besides, Cu-Sb based absorber displays good resistance to temperature and moisture [69].

### 4.5. Antimony Halide Perovskite

Antimony (Sb) is also suitable for lead free absorber layer perovskite. Sb^3+^ has an effective ionic radius of 76 pm [23] which is smaller compared to Pb^2+^ (119 pm) and Sn^2+^ (110 pm). Antimony based perovskites have a typical formula A_3_Sb_2_X_9_. Where A, is an organic cation, and X (Cl, Br, I) [8]. Photovoltaic properties of tin iodide have been intensively investigated within the last couple of years, halide complexes of antimony remained unexplored. Bromoantimonates mixed valence complexes containing both Sb (III) and Sb (V) were first reported with PCE close to 4% [70]. Regular structure (n-i-p) shows better photovoltaic performance for antimony halide perovskite as compared with the inverted structure (p-i-n). Reference [71] investigated and compared regular TiO_2_/(CH_3_NH_3_)_3_Sb_2_I_9_/spiro-OMeTAD) and inverted NiO/(CH_3_NH_3_)_3_Sb_2_I_9_/PCMB) structures and found that the regular structure performed better with PCE of 0.08%. Chlorine incorporation is a useful tactic to restrain 0-D pattern and stabilize the 2-D phase structure of (CH_3_NH_3_)_3_Sb_2_Cl_x_I_9−x_. The Sb-based absorber layer recorded PCE of 2.0% [72]. Use of sulfur and iodide as anions in MASbSI_2_ light harvester, allow the incorporation of 3+ and 4+ cations to replace 2+ inorganic cations. Power conversion efficiency (PCE) of 3.08% is realized using MASbSI_2_ [73]. Predicted results using Density Functional Theory (DFT) calculations shown that the band gap of the mixed-metal perovskite (CH_3_NH_3_)_2_AgSbI_6_ is 2.0 eV, which is confirmed by experimental results in which 1.93 eV is obtained for MA_2_AgSbI_6_. Furthermore, the device displays good stability in ambient air [74]. The reported MA_2_AgSbI_6_ perovskites exhibit low PCE. This might be due to its poor surface morphology with many pinholes. Reference [75] optimized the MA_2_Sb_2_I_9_ by emp-loying hydroiodic (HI)-chlorobenzene (CB) anti solvent to speed up heterogeneous nucleation of the Sb-based perovskite and also incorporated hydroiodic (HI)-chlorobenzene (CB) inter-layer to minimize the number of vacancies and advance quality of the inverted structure; ITO/PEDOT:PSS/perylene/HI-CB-(CH_3_NH_3_)_3_Sb_2_I_9_)/PC_70_BM/C_60_/BCP/Al. The fabricated device achieved champion power conversion efficiency (PCE) of 2.77%.

## 5. Anions Substitutions

In the organic-inorganic halide perovskite chemical composition ABX_3_, X is an anion in the formula and are usually halogens (F^−^, Cl^−^, Br^−^ and I^−^) [76], [HCOO]^−^, [CN]^−^, [N_3_]^−^, [BH_4_]^−^), SCN^−^ or mixture of them [6,13,31,76]. X anions maintained the charge neutrality between cations and anions [8]. Halogens are highly electronegative and reactive. DFT calculations indicate that the band gap is a function of the geometrical average of electro-negativities, that is, increasing the electro-negativity increases the band gap of the material [40]. One of the unique properties of lead free organic-inorganic halide perovskite is the simple way of altering the structural composition by either changing the A sites or X-site to enhance the photovoltaic performance. Halide ions migration has great impact in determining the overall photovoltaic performance [77]. Modifying the band gaps of organo-metal halide perovskites through halide substitutions may lead to instability of the material for some compositions [78,79]. Halogens (more particularly Cl^−^, Br^−^ and I^−^)) were intensively investigated due to their appropriate effective ionic radii and resulting enhancing the stability as well as the photovoltaic performance. The lead-free tin-iodide based CH_3_NH_3_SnI_3_ light absorbers have been widely synthesized and reported by [18,19,80,81,82] and shown power conversion efficiency (PCE) below 9% except for [51] recently achieved 9.0% using FASnI_3_. This is the recently reported lead-free perovskite with the highest PCE. Generally, the results indicate that iodide contributed significantly in the formation of almost regular octahedral but shows poor moisture resistance [45]. In addition, it was observed that in the Sn-based perovskite, the bandgap increases linearly with I_3_, Br_3_, and Cl_3_ [40]. It was also found that the valence band maximum of MASnI_3_ is lower than the MASnCl_3_, and conduction band maximum of MASnCl_3_ is higher than the MASnI_3_, resulting to a larger band gap of MASnCl_3_ [83]. The iodide, I^−^ (220 pm) is replaced by bromide, Br^−^ (196 pm), Cl^−^ (181 pm), or mixed halides owing similar chemical properties and proved improvement in certain compositions. Nevertheless, formation of cubic perovskite is hindered using Br^−^ (196 pm), Cl^−^ (181 pm) due to their smaller ionic radius compared to I^−^ (220 pm) [30]. Organic-inorganic perovskite solar cells are potential candidates for achieving power conversion efficiency (PCE) above 20% [3]. It is believed that organic-inorganic mixed halide perovskite is more efficient than organic-inorganic halide perovskite. This is proved by [84] in tin-based perovskite, CH_3_NH_3_SnI_3-x_Br_x_in which 5.73% power conversion efficiency (PCE) is achieved higher than the previous reported PCE using CH_3_NH_3_SnI_3_. Chlorine additive significantly helped in achieving layered phase structure, restrained the unwanted 0D dimer phase and resulted high quality (CH_3_NH_3_)_3_Sb_2_Cl_x_I_9−x_ [72]. In addition, excess chlorine in CH_3_NH_3_SnCl_3_ found positively influenced the morphological growth of the film, suppressed the oxidation of Sn^2+^ to 4+ in oxygen, and also maintained same chemical atmosphere as bulk [85]. Pseudohalogen anions with similar chemical properties and ionic radius to halogens can also influence the tolerance factor t of perovskite [31]. Thiocyanate (SCN^−^) anion is a stable pseudohalogen with ionic radius 215 pm–220 pm compared to 220 pm of I^−^ [86] can replace iodide ion in lead free perovskite. It is suggested that pseudohalogens such as CN^−^, OCN^−^, and SeCN^−^ could be promising substitutes in X component of future organic-inorganic halide perovskite solar cells [31].

## 6. Conclusions

This review discussed recent advancements on the impact of A-organic cations, B-inorganic cation, and X-anion substitutions on stability, efficiency, and photovoltaic performance of lead-free organic-inorganic halide perovskite. Generally, it concludes that structural modification has great impact on the bandgap and the overall photovoltaic performance of the perovskite solar cells.

Dimensionality and stability of perovskites lattice could be significantly affected by the size of A-cation. It is observed that stability against moisture was remarkably enhanced by substituting FA^+^ by Cs^+^ and mixing MA and FA improved the morphology and reduce the charge carrier recombination of the perovskites. A-cation substitution was found to be responsible for expansion, contraction, or octahedral deformation of the perovskite framework and this could also directly or indirectly influence the bandgap and optical characteristics of the material. Increase in the ionic radius resulted in the increase in bandgap and octahedral deformation in lead-free perovskite solar cells.

Generally, from all the reported lead-free perovskite, Sn-halide perovskite is the champion, efficient and promising candidate to compete with lead based perovskite. Theoretical results predict that 24.82% PCE could be achieved using tin as a substitute of toxic divalent lead metal. Recently PCE of 9.0% was reported in tin halide perovskite which is the highest PCE achieved in lead-free based organic-inorganic halide perovskite solar cells. Instability associated with tin halide is the major issue concerning tin-based perovskite. Understanding the process of self doping is required in designing strategies towards achieving long term stability in tin-based perovskite. Recently [87] suggested an immense research on roles on solvent precursors, film thickness, temperature and growth condition, humidity and reductive additives as a future direction to overcome instability in perovskite solar cells. Another strategy to further improve the overall photovoltaic performance is by employing different kinds of photovoltaic polymers and low band gap polymers on top of perovskite [88,89].

Germanium is also found to be a potential divalent metal for replacing lead. Ge^2+^ is chemically unstable. Ge-based perovskite was reported with wide bandgap >1.6 eV and very low PCE of ≤0.57%. This might be due to its smaller ionic radius (73 pm) and poor solubility in the polar solvent.

Bismuth, Bi^3+^ is an iso-electronic to Pb^2+^ having the same 6s^2^ lone pair and has effective ionic radius equivalent to Pb^2+^ (Bi^3+^ = 103 pm and Pb^2+^ = 119 pm). Bismuth attracted attention due to its non-toxic nature, ambient stability, and simple solution method processing. Bismuth halide perovskite suffered from too large bandgap resulted in very low PCE of <0.3%.

Transition metals such as Cu in particular have appropriate oxidation state to replace lead, although, the corner sharing network of halide octahedral may be compromised due to their smaller ionic radius (Cu^+^ = 77 pm, Cu^2+^ = 73 pm). Cu^2+^ is more stable than Sn^2+^ and Ge^2+^. The new absorber Rb_2_(CuIn)Cl_6_ shows a required band gap of 1.36 eV, strong light absorption. The highest PCE achieved in Cu halide perovskite is 0.63%, although, PCE of 11.2% is obtained for Cu_2_ZnSn(Se,S) [90].

Antimony is another alternative to lead due to its good opto-electronic properties and ability to form in different structural dimension ranging from zero dimensional dimer to 3-dimension. PCE of 2.77% was reported in Sb-halide perovskite with poor surface morphology with many pinholes.

Halide ions play a major role in maintaining the charge neutrality between cations and anions. Halogen with lower electronegativity is essential in achieving appropriate bandgap because bandgap increases linearly with I, Br, Cl. Mixing two different halides was found to be more efficient. Pseudohalogens with similar chemical properties with real halogens are potential components in future lead-free organic inorganic perovskite solar cells.

## Figures and Tables

**Table 1:** Calculated effective ionic radii of A-organic cations, metal cations and halide anions [10,13,22,23].

A-Cation	$r_{A,eff}$/pm	Ref.	B-Cation	$r_{B,eff}$/pm	Ref.	X-Anion	$r_{X,eff}$/pm	Ref.
Ammonium [NH_4_]^+^	146	[13]	Be^2+^	45	[22]	I^-^	220	[10]
Hydroxylammonium [NH_3_OH]^+^	216	[13]	Mg^2+^	72	[22]	Br^-^	196	[10]
Methylammonium [CH_3_NH_3_]^+^	217	[13]	Ca^2+^	100	[22]	Cl^-^	181	[10]
Hydrazinium [NH_3_NH_2_]^+^	217	[13]	Ti^2+^	86	[22]	F^-^	133	[22]
Azetidinium [(CH_2_)_2_NH_2_]^+^	250	[13]	V^2+^	79	[22]			
Formadinium [CH(NH_2_)_2_]^+^	253	[13]	Cr^2+^	80	[22]			
Imidazoline [C_3_N_2_H_5_]^+^	258	[13]	Mn^2+^	83	[22]			
Ethylammonium [CH_3_CH_2_)NH_3_]^+^	274	[13]	Fe^2+^	78	[22]			
Guanidinium [(NH_2_)_3_C]^+^	278	[13]	Ni^2+^	69	[22]			
Tetramethylammonim [(CH_3_)_4_N]^+^	292	[13]	Cu^1+^	77	[22]			
Thiazolium [C_3_H_4_N_5_]^+^	320	[13]	Cu^2+^	73	[22]			
3-pyrollinium [NC_4_H_8_]^+^	272	[13]	Zn^2+^	74	[22]			
Tropylium [C_7_H_7_]^+^	333	[13]	Ge^2+^	73	[22]			
Cesium [Cs]^+^	167	[22]	Sr^2+^	118	[22]			
Potassium [K]^+^	138	[22]	Ag^2+^	94	[22]			
			Ag^1+^	115	[22]			
			Cd^2+^	95	[22]			
			Ba^2+^	135	[22]			
			Eu^2+^	117	[22]			
			Tm^2+^	103	[22]			
			Yb^2+^	102	[22]			
			Pt^2+^	80	[22]			
			Hg^2+^	102	[22]			
			Np^2+^	110	[22]			
			Ni^2+^	69	[22]			
			Sn^2+^	115	[10]			
			Sb^3+^	76	[23]			
